# Application of nested multiplex polymerase chain reaction respiratory and pneumonia panels in children with severe community‐acquired pneumonia

**DOI:** 10.1002/jmv.28334

**Published:** 2022-12-02

**Authors:** Ting‐Yu Yen, Jian‐Fu Chen, Chun‐Yi Lu, En‐Ting Wu, Ching‐Chia Wang, Frank Leigh Lu, Li‐Min Huang, Luan‐Yin Chang

**Affiliations:** ^1^ Department of Pediatrics, National Taiwan University Hospital, College of Medicine National Taiwan University Taipei Taiwan

**Keywords:** children, community acquired infection, intensive care units, nested multiplex PCR, pneumonia

## Abstract

Community‐acquired pneumonia (CAP) is a serious clinical concern. A lack of accurate diagnosis could hinder pathogen‐directed therapeutic strategies. To solve this problem, we evaluated clinical application of nested multiplex polymerase chain reaction (PCR) in children with severe CAP. We prospectively enrolled 60 children with severe CAP requiring intensive care between December 2019 and November 2021 at a tertiary medical center. Nested multiplex PCR respiratory panel (RP) and pneumonia panel (PP) were performed on upper and lower respiratory tract specimens. We integrated standard‐of‐care tests and quantitative PCR for validation. The combination of RP, PP, and standard‐of‐care tests could detect at least one pathogen in 98% of cases and the mixed viral‐bacterial detection rate was 65%. The positive percent agreement (PPA), and negative percent agreement (NPA) for RP were 94% and 99%; the PPA and NPA for PP were 89% and 98%. The distribution of pathogens was similar in the upper and lower respiratory tracts, and the DNA or RNA copies of pathogens in the lower respiratory tract were equal to or higher than those in the upper respiratory tract. PP detected bacterial pathogens in 40 (67%) cases, and clinicians tended to increase bacterial diagnosis and escalate antimicrobial therapy for them. RP and PP had satisfactory performance to help pediatricians make pathogenic diagnoses and establish therapy earlier. The pathogens in the upper respiratory tract had predictive diagnostic values for lower respiratory tract infections in children with severe CAP.

## 1 INTRODUCTION

Pneumonia is a serious common disease worldwide. For children under the age of five, it has been estimated that approximately 900 000 children died of pneumonia in 2015 globally, accounting for 15% of child mortality.^1^ Severe community‐acquired pneumonia (CAP), defined as CAP requiring admission to an intensive care unit (ICU),^2^ is associated with high morbidity and mortality. Compared with adults, children have more mixed viral‐viral or viral‐bacterial pathogens.^3^, ^4^, ^5^ Mixed infection may greatly increase the disease severity and hospitalization rates.^3^, ^4^, ^5^

In clinical practice, diagnostic tests are mainly based on standard‐of‐care (SOC) tests, such as microbial cultures, serological detection, and fluorescent immunoassays, which are relatively time‐consuming and have low sensitivity. In addition, the diagnostic rate is limited due to prior antibiotic usage and the difficulty in collecting high‐quality lower respiratory tract specimens, particularly in young children who are often uncooperative. The lack of an accurate microbial diagnosis might hinder well‐established pathogen‐directed treatment plans. Presumably, lower respiratory tract infections in children usually result from the replication and spread of pathogenic viruses and bacteria from the upper respiratory tract, which might invade the mucosa and lower airways, resulting in clinical inflammation. Therefore, it is worthwhile to verify whether upper respiratory tract pathogens could predict lower respiratory tract infection in young children, among whom it is difficult to obtain high‐quality lower respiratory tract specimens.

Currently, the development of nucleic acid amplification detection has rapidly promoted the identification of viral or microbial pathogens, which is of great significance for the treatment and control of infection.^6^ By combining SOC diagnostic tests with molecular diagnostics, such as multiplex polymerase chain reaction (PCR), the diagnostic rate could reach approximately 80% for CAP in children.^7^ Recently, highly automated nested multiplex PCR‐based syndromic assays have been developed and can detect multiple pathogens simultaneously within a laboratory turnaround time of only approximately one hour.^8^, ^9^ Early detection of multiple pathogens allows for timely development of appropriate antibiotic strategies and might therefore improve clinical outcomes in adults.^10^, ^11^ However, few studies have been conducted in the pediatric population, specifically for severe CAP. This prospective cohort study thus aimed to evaluate the performance and clinical impact of nested multiplex PCR panel‐based molecular diagnostic approach and to compare pathogens and their DNA or RNA copies for upper and lower respiratory tract specimens of severe CAP in children.

## 2 METHODS

### 2.1 Study design and patient enrollment

Between December 2019 and November 2021, we prospectively enrolled children (under 18 years of age) with severe CAP, defined as CAP requiring admission to pediatric ICUs at National Taiwan University Hospital (NTUH), a 2600‐bed tertiary medical center. The inclusion criteria were based on clinical evidence of acute lower respiratory tract infection and positive radiological evidence of pneumonia on chest X‐ray (CXR) or computed tomography (CT) within 48 h after admission (Supporting Information: Table 1). Children diagnosed with aspiration pneumonia, defined as massive aspiration of oropharyngeal or upper gastrointestinal contents associated with pneumonia, were excluded.^12^ Cases for whom nosocomial infection could not be ruled out (defined as last admission less than 28 days ago) were also excluded.^13^ The patients were further divided into two groups: critical cases and severe cases. Critical cases were defined as CAP patients with septic shock or respiratory failure requiring endotracheal ventilator support. Severe cases were CAP patients who required ICU admission without septic shock or endotracheal ventilator support.

### 2.2 Ethics approval statement and patient consent statement

This study was approved by the National Taiwan University Hospital Research Ethics Committee, and the Institutional Research Board number was 201907108RINB. Informed consent was obtained from the patients and their parents or guardians.

### 2.3 Clinical specimens and data collection

Acute‐phase upper respiratory tract specimens, such as nasopharyngeal swabs, and lower respiratory tract specimens, such as induced sputum, endotracheal aspirate (ETA), or bronchoalveolar lavage (BAL), were collected by trained staffs from all enrolled children within 48 h after admission. All respiratory samples were transported to the clinical microbiology and virology laboratories of NTUH, which were accredited by the College of American Pathologists and the Taiwan Accreditation Foundation, for SOCdiagnostic tests such as microbial cultures, serological tests, fluorescent immunoassays, and PCR. Clinical characteristics, results of SOC diagnostic tests, and electronic medical record (EMR) data were collected for enrolled children.

## 3 MULTIPLEX PCR PNEUMONIA PANEL AND RESPIRATORY PANEL

FilmArray® BioFire® Pneumonia Panel (PP) and Respiratory Panel 2.1 (RP) are syndrome specific, cartridge‐based, nested multiplex PCR performed in an automated manner with results available in approximately 1 h (bioMérieux).^14^, ^15^ RP tests for 15 viral and 4 bacterial respiratory pathogens with qualitative results (Supporting Information: Table 2). PP provides semiquantitative results for 15 bacteria and qualitative results for eight viruses, three atypical bacteria and seven antimicrobial resistance genes (Supporting Information: Table 3). To compare the diagnostic performance and pathogen distributions between upper and lower respiratory tract specimens, we performed the RP on nasopharyngeal swabs and the PP on induced sputum, ETA, or BAL in parallel for each case by trained laboratory technicians following the manufacturer's instructions.

### 3.1 Orthogonal validation

To orthogonally validate the results of the PP and the RP, two strategies were employed. First, respiratory specimens were sent for Gram staining and culture to detect common culturable pathogens according to standard protocols. Second, for difficult‐to‐cultivate microorganisms, real‐time quantitative PCR (qPCR)‐based nucleic acid detection was applied. The methods and primer sets for nine viruses and four bacteria are described in Supporting information: Table 4.

### 3.2 Study endpoints and statistical analysis

As the primary study endpoint, to evaluate the diagnostic performance of this novel diagnostic technology, pathogen detection rates, positive percent agreement (PPA), and negative percent agreement (NPA) were assessed. To compare the pathogen detection rates between the nested multiplex PCR panels and SOC diagnostic tests, the proportion of pathogen types was calculated by *χ* ^2^ analysis. The PP and RP were considered concordant, such as true positive (TP) or true negative (TN), when they were consistent with the results from the corresponding orthogonal validation tests. Microorganisms identified only by RP or PP and not by the orthogonal validation tests were considered false‐positives (FP), and vice versa were considered false negatives (FN). The diagnostic agreement between the multiplex PCR panels and orthogonal validation tests was measured for each pathogen in the form of positive percent agreement (PPA = TP/[TP + FN]), negative percent agreement (NPA = TN/[TN + FP]), and overall percent agreement (OPA = [TP + TN]/[TP + FP + TN + FN]).^8^

As the secondary endpoint, pathogens and their DNA or RNA copies were compared between the upper and lower respiratory tracts. The distribution and concordance rates of pathogens in the upper and lower respiratory tracts were assessed. To assess the efficiency of pathogen detection for upper and lower respiratory tract specimens, the McNemar test was used. To evaluate the DNA or RNA copies of pathogens from qPCR between the upper and lower respiratory tract, the Wilcoxon signed rank test was used.

For the analysis of clinical outcome, the pediatric sequential organ failure assessment score (pSOFA score) was used to grade organ dysfunction in pediatric patients.^16^ For categorical data, *χ* ^2^ tests or Fisher's exact test was used to measure the difference. The Shapiro–Wilk test was used to test the distribution of continuous variables. Mean (standard deviation) and *t*‐tests were used for data with a normal distribution, and median (interquartile range) and Mann–Whitney U tests were used for data with a non‐normal distribution. A multivariable analysis was performed to identify the most significant factors associated with the clinical outcome or severity. *p* values less than 0.05 were considered statistically significant. SPSS (version 24) was used for statistical analysis.

## 4 RESULTS

### 4.1 Demographic data and clinical characteristics

From December 2019 to November 2021, a total of 60 children with severe CAP admitted to the ICU were enrolled. The patients were divided into critical cases (30/60, 50%) and severe cases (30/60, 50%). Their demographic data and characteristics are shown in Table 1. Anemia, lower hemoglobin (Hb) level, lung consolidation, procalcitonin higher than 0.5 ng/ml, and pSOFA scores were significantly more frequent or higher in the critical case group than in the severe case group. In multivariable analysis, the most significant factors associated with critical cases were Hb level [adjusted odds ratio (OR), 0.4, 95% confidence interval (CI), 0.2–0.7, *p* < 0.01] and pSOFA score (adjusted OR, 2.5, 95% CI, 1.5–4.2, *p* < 0.01), adjusted for age and sex.

**Table 1:** Clinical characteristics and laboratory results at admission of children with community‐acquired pneumonia requiring intensive care

Demographics	Total cases (*N* = 60)	Critical case (*N* = 30)	Severe case (*N* = 30)	*p* Value
Male/female (ratio)	37/23 (1.6)	18/12 (1.5)	19/11 (1.7)	0.79
Age, median (IQR)	1.3 (0.6–3.4)	1.1 (0.5–4.2)	1.4 (0.7–3.2)	0.48
Antimicrobial usage	23 (38)	14 (47)	9 (30)	0.18
Cluster/contact history	27 (45)	12 (40)	15 (50)	0.44
Prodrome (days)	3 (1–5.5)	3 (1–7)	2 (1–7.5)	0.90
Comorbidities—*N* (%)	43 (72)	21 (70)	22 (73)	0.77
Chest or airway diseases	20 (33)	8 (27)	12 (40)	0.27
Preterm birth	17 (28)	7 (23)	10 (33)	0.39
Cardiovascular diseases	20 (33)	10 (33)	10 (33)	>0.99
Other diseases	20 (33)	14 (47)	6 (20)	0.03
Symptoms—*N* (%)				
Fever	58 (97)	29 (97)	29 (97)	>0.99
Cough	56 (93)	26 (87)	30 (100)	0.11
Dyspnea	51 (85)	24 (80)	27 (90)	0.47
Cyanosis	41 (68)	23 (77)	18 (60)	0.17
Rhinorrhea	26 (43)	15 (50)	11 (37)	0.30
General malaise	28 (47)	12 (40)	16 (53)	0.30
Physical examination				
Hypotension	18 (30)	18 (60)	0 (0)	<0.001
Tachycardia	32 (53)	21 (70)	11 (37)	0.01
Tachypnea	55 (91.7)	26 (87)	29 (97)	0.35
Desaturation	45 (75)	25 (83)	20 (67)	0.14
Temperature, °C	38.6 (38.2–39.5)	38.8 (38.3–39.7)	38.5 (38.2–39.2)	0.37
Laboratory results				
Leucocytes (x10^3^/µl)	12.6 (9.3–16.8)	14.1 (9.1–18.8)	12.3 (9.1–15.6)	0.20
Hb, g/dL (Mean ± SD)	12.3 ± 2.3	11.4 ± 2.7	13.1 ± 1.5	0.003
Anemia	7 (12)	7 (23)	0 (0)	0.01
Platelets, K/μl	331 (254–446)	356 (271–467)	315 (243–433)	0.33
hsCRP > 1 mg/dl	30 (50)	16 (53)	14 (47)	0.61
PCT > 0.5 ng/ml	20 (33)	14 (47)	6 (20)	0.03
Radiography—*N* (%)				
Consolidation	26 (43)	18 (60)	8 (27)	0.01
Interstitial infiltrate	59 (98)	30 (100)	29 (97)	>0.99
Pleural effusion	12 (20)	4 (13)	8 (27)	0.33
pSOFA score	3 (2–6)	6 (3–8)	2 (1–3)	<0.001

*Note*: Data are shown as the median (IQR), mean (±SD), or number (%).

Abbreviations: Hb, hemoglobin; hsCRP, high‐sensitivity C‐reactive protein; IQR, interquartile range; *N*, number of patients; PCT, procalcitonin; pSOFA score, pediatric sequential organ failure assessment score; SD, standard deviation.

### 4.2 Diagnostic performance

**Figure 1:**
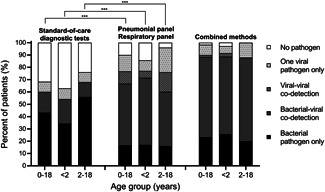
Proportion of pathogen types by different diagnostic methods for children with severe community‐acquired pneumonia. The proportion of pathogen types in 60 children with severe community‐acquired pneumonia admitted to the pediatric intensive care unit through different diagnostic methods from December 2019 to November 2021. Standard‐of‐care diagnostic tests including microbial cultures, serological testing, fluorescent immunoassays, and polymerase chain reaction (PCR). Pneumonia panel and Respiratory panel denote the FilmArray® BioFire® Pneumonia Panel and Respiratory Panel 2.1, which are syndrome‐specific, cartridge‐based, nested multiplex PCRs. Combined methods means the integration of standard of care diagnostic tests, pneumonia panels and respiratory panels. ***Statistical difference, *p* < 0.01.

As shown in Figure 1, the detection rate of at least one potential pathogen by the SOC diagnostic tests was 68%. The detection rates of at least one potential pathogen by PP alone or RP alone were 87% and 78%, respectively. Furthermore, the detection rate of at least one potential pathogen by combined PP and RP significantly increased to 90% compared with the SOC diagnostic tests (*p* < 0.01). After integration of the SOC diagnostic tests, PP and RP, the detection rates of at least one potential pathogen noticeably increased to 98%, and the mixed viral‐bacterial detection rate increased to 65%.

**Figure 2:**
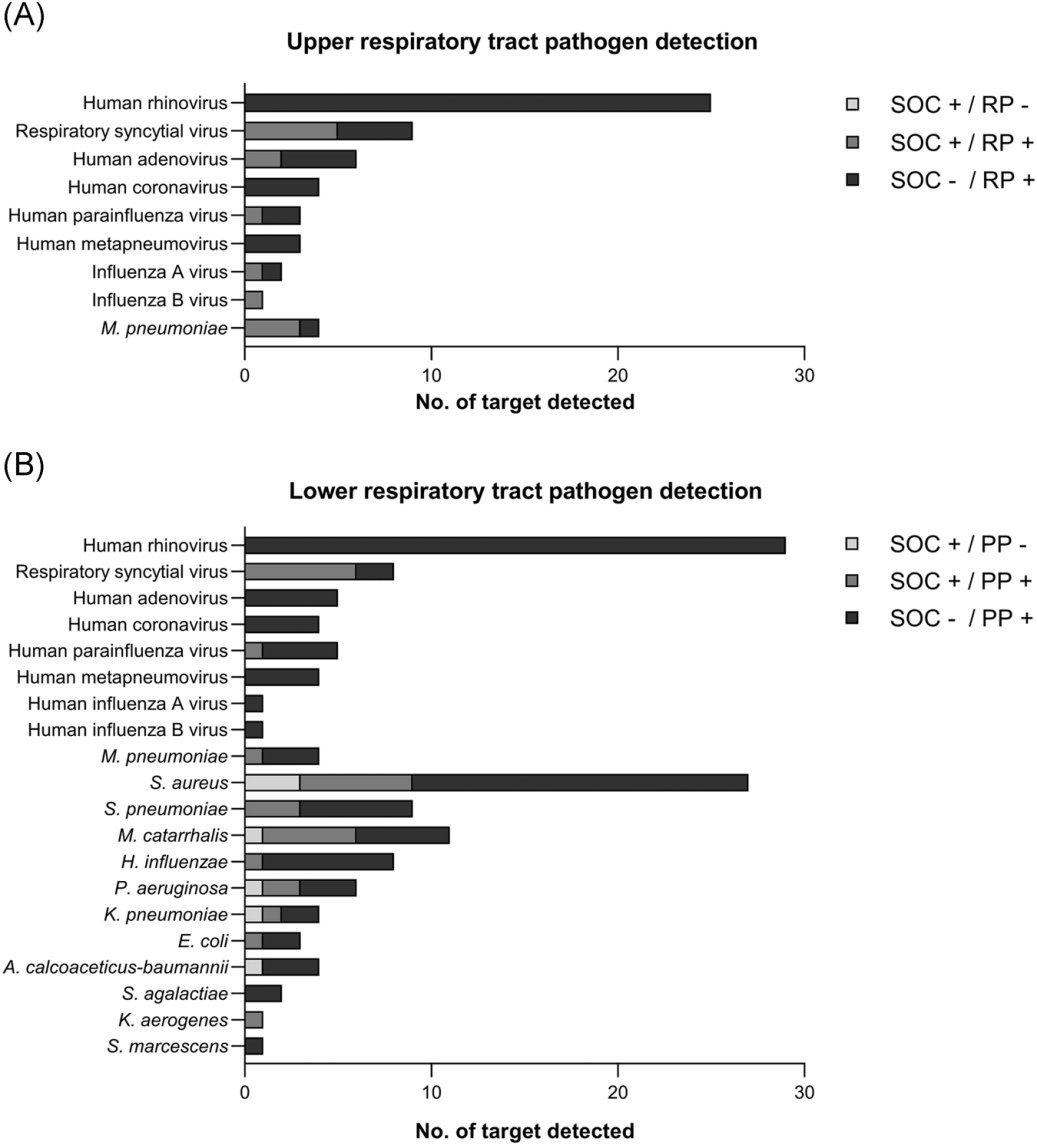
Pathogen distribution in the FilmArray Respiratory Panel or the Pneumonia Panel compared with standard‐of‐care diagnostic methods in children with severe community‐acquired pneumonia. (A) Pathogen distribution in the FilmArray Respiratory Panel compared with the standard‐of‐care diagnostic methods of upper respiratory specimens. (B) Pathogen distribution in the FilmArray Pneumonia Panel compared with the standard‐of‐care diagnostic methods of the lower respiratory tract specimens.  PP, pneumonia panel; RP, respiratory panel; SOC, standard‐of‐care diagnostic tests.

As shown in Figure 2, regardless of RP in upper respiratory tract specimens or PP in lower respiratory tract specimens, the nested multiplex PCR technique could detect all cases of common respiratory viruses and atypical bacteria. In the lower respiratory tract, PP could detect most bacteria (70/77, 91%) while only seven of them were detected by SOC diagnostic tests. Overall, the three most common respiratory viruses were human rhinovirus, respiratory syncytial virus, and adenovirus. Human rhinovirus was detected as the only pathogen in 6 cases with severe CAP. The three most common respiratory bacteria were *Staphylococcus aureus, Streptococcus pneumoniae*, and *Moraxella catarrhalis*. Four cases with *S. aureus, M. catarrhalis*, or *Pseudomonas aeruginosa* were not detected in the initial cultures but were positive in the subsequent microbial cultures several days after the PP test.

After integration of the SOC diagnostic test and qPCR test into the orthogonal validation test, the PPA and NPA of RP and PP were between 90% and 100% for most viruses and *Mycoplasma pneumoniae*, except human metapneumovirus and influenza B virus (Supporting Information: Tables 5 and 6). For *S. aureus*, *S. pneumoniae*, and *Haemophilus influenzae*, the OPAs between the PP and orthogonal validation tests were over 90%. For all analytes of nine viruses and four bacteria, the PPA and NPA of RP were 94% and 99%, and the PPA and NPA of PP were 89% and 98%, respectively.

### 4.3 Comparison of pathogens between upper and lower respiratory tracts

To elucidate the relationship between the upper and lower airways, we integrated PP, RP, SOC diagnostic tests and quantitative PCR to comprehensively present the pathogens detected in this study (Supporting Information: Figure 1). Overall, the pathogen distribution in the upper and lower respiratory tract was highly similar. The concordance rate of pathogens detected in upper and lower respiratory tract specimens was 96%. For severe CAP cases, the efficiency of pathogen detection of lower respiratory tract specimens was higher than that of upper respiratory tract specimens (*p* < 0.001).

**Figure 3:**
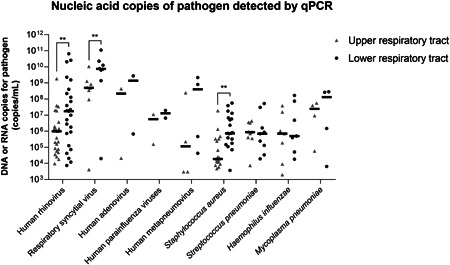
Comparison of DNA or RNA copies of pathogens between upper and lower respiratory tract specimens of 60 children with severe community‐acquired pneumonia. **Statistical difference, *p* < 0.05. qPCR, real‐time quantitative polymerase chain reaction.

Furthermore, we analyzed paired qPCR DNA or RNA copies of five viruses, three bacteria, and one atypical bacterium in upper and lower respiratory tract specimens (Figure 3). Overall, the nucleic acid copies of the lower respiratory tract specimens from the same case generally tended to be higher than those of the upper respiratory tract specimens. In particular, the nucleic acid copies of human rhinovirus, respiratory syncytial virus and *S. aureus* in the lower respiratory tracts of severe CAP patients were significantly higher than those in the upper respiratory tracts (*p* values were 0.001, 0.008, and 0.006, respectively).

### 4.4 Clinical impact and outcome

In an analysis of clinical outcomes, 27 of 30 critical cases (90%) required endotracheal ventilator support. Therefore, the specimen types were ETA in 27 cases and induced sputum in 33 cases. From the results of PP, more bacteria (27/33 vs. 13/27, *p* = 0.01), viruses (27/33 vs. 13/27, *p* = 0.01) or mixed viral and bacterial pathogens (22/33 vs. 6/27, *p* < 0.01) were detected in induced sputum than in ETAs. In 40 cases with bacterial pathogens detected in PP, clinicians were more likely to make the diagnosis of bacterial infection (25/40 vs. 0/20, *p* < 0.01), escalate antimicrobial treatment (11/40 vs. 0/20, *p* = 0.01), and increase infection control policies (9/40 vs. 0/20, *p* = 0.02) than in 20 cases without bacteria detected in PP. Among all 60 enrolled cases, three cases required surgical intervention due to empyema, and two cases died.

## 5 DISCUSSION

This study reported the performance of the combined RP, PP, and SOC diagnostic tests for detecting at least one pathogen in 98% of cases of severe CAP. We also found that the distribution of pathogens in the upper and lower respiratory tracts was similar with a high concordance rate, and the DNA or RNA copies of pathogens in the lower respiratory tracts were similar to or higher than those in the upper respiratory tracts. When bacterial pathogens were detected in PP, clinicians tended to increase the diagnosis of bacterial infection, escalate antimicrobial therapy, and increase infection control strategies compared with cases in which bacteria were not detected.

Based on real‐world data from previous clinical studies, the detection rates of at least one pathogen for RP in respiratory tract infections in children ranged from 48% to 80%.^17^, ^18^ There have been no published studies of PP focused on children. For the real‐world experience of PP in adults, the detection rates of at least one pathogen ranged from 58% to 91% in lower respiratory tract infections.^9^, ^19^ Gilbert et al. conducted a parallel study of RP, PP, and SOC diagnostic tests for CAP in adults. The detection rates of at least one pathogen were 81% and 91% for the RP combined with SOC tests and PP combined with SOC tests, respectively.^20^ In this prospective cohort study of children with severe CAP, PP or RP alone could detect at least one potential pathogen in 87% and 78% of cases, respectively. After integration of the SOC diagnostic tests, PP, and RP, the detection rate of at least one potential pathogen dramatically increased to 98%, and the mixed viral‐bacterial detection rate increased to 65% in children with severe CAP. The diagnostic performance of our study was generally better than previous studies in pediatric and adult groups.^8^, ^15^, ^17^, ^18^, ^21^

In children, lower respiratory tract infections might be induced by pathogenic viruses and bacteria in the upper respiratory tract. Teo S.M., et al. reported that the nasopharyngeal microbiome in infants could impact the severity of lower respiratory infection.^22^ In addition, a respiratory microbiome study based on qPCR and 16S rRNA sequencing could serve as a valid proxy for lower respiratory tract microbiota in childhood pneumonia, which can help to establish diagnostic and treatment strategies.^23^ Our study showed that the concordance rate of pathogens detected in the upper and lower respiratory tract specimens was high (96%), and the correlation of pathogen titers was good. When available, lower respiratory tract specimens are more diagnostically efficient than upper respiratory tract specimens (*p* < 0.001). In the real world, however, obtaining high‐quality lower respiratory tract specimens from children with lower respiratory tract infections is difficult. Therefore, the use of upper respiratory tract specimens combined with highly sensitive pathogenic molecular detection techniques might be a potential diagnostic solution.

We found that clinicians tended to adjust their clinical diagnosis and management strategies when bacterial pathogens were detected in PP. Some bacteria grew in subsequent microbial cultures several days after the PP test. These findings suggest that initial detection by nucleic acid detection techniques such as PP or qPCR might have significant clinical implications in children, especially those suffering from severe CAP.

In our study, 53% (32/60) of children had human rhinovirus detected in their lower respiratory tract specimens, and six children had human rhinovirus (hRV) as the only detected pathogen. In addition, hRV qPCR RNA copies were significantly higher in lower respiratory tract specimens than in upper respiratory tract specimens. Human rhinovirus is the most common cause of respiratory diseases in children, accounting for more than half of acute upper respiratory tract infections,^24^ and is increasingly being linked to severe respiratory diseases, especially since molecular diagnostics have been developed.^25^, ^26^, ^27^, ^28^ During the COVID‐19 pandemic, it has been observed that SARS‐CoV‐2‐positive patients coinfected with rhinovirus might be at similar or higher risk for increased clinical severity.^28^, ^29^ Therefore, hRV found in the lower respiratory tract should no longer be viewed as a bystander only.

## 6 LIMITATIONS

The strength of our study was the prospective combination of FilmArray RP and PP for the detection of upper and lower respiratory tract pathogens in pediatric ICU patients, but there were some limitations. First, the study period coincided with the COVID‐19 pandemic. Due to social distancing and wearing masks, the prevalence of influenza and other respiratory viruses decreased, and the characteristics of infections during epidemics might be different from those during the non‐epidemic period. Second, we designed qPCR orthogonal validation experiments for most of the viruses and bacteria covered by PP, but there were some viruses and bacteria with a low prevalence, and these pathogens were not included in the analysis. Third, this was a prospective single‐center observational cohort study, and a multicenter randomized controlled study is required to verify the potential value of its clinical application in the future.

## 7 CONCLUSION

Our study demonstrated that a new molecular diagnostic technique, nested multiplex PCR RP and PP, had powerful diagnostic performance and could help clinicians make pathogenic diagnoses and start specific antimicrobial therapy in a timely manner. Since lower respiratory tract specimens are difficult to obtain, upper respiratory tract specimens combined with molecular diagnostics might break the diagnostic barrier in children.

## AUTHOR CONTRIBUTIONS

Ting‐Yu Yen and Luan‐Yin Chang developed the idea, designed the study, and were responsible for the accuracy of the data analysis. Yen‐Yu Yen and Jian‐Fu Chen conducted experiments, collected and analyzed data, and created graphs and tables. Chun‐Yi Lu, En‐Ting Wu, Ching‐Chia Wang, and Frank Leigh Lu enrolled study patients and collected clinical specimens. Ting‐Yu Yen, Jian‐Fu Chen, and Luan‐Yin Chang wrote the manuscript. Li‐Min Huang and Luan‐Yin Chang provided advice and suggestion for improving the manuscript. All authors reviewed and approved the final manuscript.

## CONFLICT OF INTEREST

The authors declare no conflict of interest.

## Supporting information

Supplementary information.

Supplementary information.

## Data Availability

The data that support the findings of this study are available from the corresponding author upon reasonable request.
