# Supplementary material for: Application of nested multiplex polymerase chain reaction respiratory and pneumonia panels in children with severe community‐acquired pneumonia
Source: J Med Virol. 2022 Dec 2;95(1):e28334. doi: 10.1002/jmv.28334 (PMC10108056; doi:10.1002/jmv.28334)
Supplement: Supplementary file 1 — Supplementary information. [file JMV-95-0-s001.docx]

| **Supplementary Table 1.**  **Inclusion Criteria of Severe Community-acquired Pneumonia** |
| --- |
| 1. Pediatric patients age 0-18 years old |
| 1. Admitted to pediatric intensive care unit within 48 hours |
| 1. With clinical evidences for community-acquired pneumonia |
| 1. Acute infection evidences: fever or hypothermia |
| 1. Lower respiratory tract infection evidence: cough, sputum, pleural chest pain, dyspnea, tachypnea, respiratory distress, or respiratory failure |
| 1. Pneumonia with radiologic evidences: consolidation, infiltrate, or pleural effusion on chest X-ray or chest computed tomography within 48 hours before or after admission |

| **Supplementary Table 2.**  **Targets of the FilmArray^®^ BioFire^®^ Respiratory Panel 2.1** |
| --- |
| **Viruses** |
| Adenovirus |
| Coronavirus 229E |
| Coronavirus HKU1 |
| Coronavirus NL63 |
| Coronavirus OC43 |
| Human Metapneumovirus |
| Human Rhinovirus/Enterovirus |
| Influenza A |
| Influenza B |
| Parainfluenza Virus 1 |
| Parainfluenza Virus 2 |
| Parainfluenza Virus 3 |
| Parainfluenza Virus 4  Respiratory Syncytial Virus  **Bacteria**  *Bordetella parapertussis* (IS1001) *Bordetella pertussis* (*ptxP*) *Chlamydia pneumoniae* *Mycoplasma pneumoniae* |

| **Supplementary Table 3.**  **Targets of the** **FilmArray^®^ BioFire^®^ Pneumonia Panel** |
| --- |
| **Bacteria** |
| *Acinetobacter calcoaceticus*-*baumannii* complex |
| *Enterobacter cloacae* complex |
| *Escherichia coli* |
| *Haemophilus influenzae* |
| *Klebsiella aerogene* |
| *Klebsiella oxytoca* |
| *Klebsiella pneumoniae* group |
| *Moraxella catarrhalis* |
| *Proteus spp.* |
| *Pseudomonas aeruginosa* |
| *Serratia marcescens* |
| *Staphylococcus aureus* |
| *Streptococcus agalactiae* |
| *Streptococcus pneumoniae* |
| *Streptococcus pyogenes* |
| **Atypical Bacteria** |
| *Chlamydia pneumoniae* |
| *Legionella pneumophila* |
| *Mycoplasma pneumoniae* |
| **Viruses** |
| Adenovirus |
| Coronavirus |
| Human Metapneumovirus |
| Human Rhinovirus/Enterovirus |
| Influenza A |
| Influenza B |
| Parainfluenza Virus |
| Respiratory Syncytial Virus |
| **Antimicrobial Resistance Genes** |
| CTX-M |
| IMP |
| KPC |
| mecA/C and MREJ  NDM |
| OXA-48-like |
| VIM |

| Supplementary Table 4.  Primer set of orthogonal quantitative polymerase chain reaction. | | |
| --- | --- | --- |
| Pathogen | **Forward primer**  **(5’- 3’)** | **Reverse primer**  **(5’- 3’)** |
| Human Rhinovirus | GACARGGTGTGAAGASYC | CAAAGTAGTYGGTCCCRTCC |
| Human Enterovirus | TCCTCCGGCCCCTGAATG | AATTGTCACCATAAGCAGCCA |
| Respiratory Syncytial Virus | GCAGGATTGTTTATGAATGCC | CTTCCACAACTTGYTCCATTTC |
| Adenovirus | GCCCCAGTGGTCTTACATGCACATC | GCCACGGTGGGGTTTCTAAACTT |
| Parainfluenza Virus 1 | ACCTACAAGGCAACAACATC | CTTCCTGCTGGTGTGTTAAT |
| Parainfluenza Virus 3 | GGAGCATTGTGTCATCTGTC | TAGTGTGTAATGCAGCTCGT |
| Human Metapneumovirus | CATCAGGTAATATCCCACAAAATCAG | GTGAATATTAAGGCACCTACACATAATAARA |
| Influenza A | GACCRATCCTGTCACCTCTGAC | AGGGCATTYAGGACAAAKCGTCTA |
| Influenza B | AAATACGGTGGATTAAATAAAAGCAA | CCAGCAATAGCTCCGAAGAAA |
| *Mycoplasma pneumoniae* | CCAACCAAACAACAACGTTCA | TAACGGCAACACGTAATCAGGTC |
| *Staphylococcus aureus* | GTTGCTTAGTGTTAACTTTAGTTGTA | AATGTCGCAGGTTCTTTATGTAATTT |
| *Streptococcus pneumoniae* | GCAGTACAGCAGTTTGTTGG | CAGTCCCAGTCGGTGCTGTC |
| *Haemophilus influenzae* | AATGCGTGATGCTGGTTATGAC | AAGAGTTTTGCGATAGATTCATTGG |

Primer sets for nine viruses and four bacteria were described above. Briefly, all collected respiratory specimens were immediately sent to the hospital laboratories, and upon completion of nested multiplex PCR testing, they were applied to deoxyribonucleic acid (DNA)/ ribonucleic acid (RNA) shieldsTM (Zymo, Irvine, US) and stored at -80°C. For viral detection, RNA was extracted by Quick-RNA™ Miniprep Kit (Zymo, Irvine, US) and then complementary deoxyribonucleic acid (DNA) was synthesized by SuperScript™ III Reverse Transcriptase (Thermo, Waltham, US). For microbial pathogens, microbial DNA was extracted by QIAamp DNA Microbiome Kit (QIAGEN, Hilden, Germany). The fluorescence of detection was obtained from Roche LightCycler™ 2.0 (Roche, Basel, Switzerland), and absolute quantification for each pathogen was calculated from standard curve generated from plasmid.

**Supplementary Table 5. Performance summary of the pathogens detected by the Filmarray respiratory panel compared to standard-of-care testing and quantitative PCR of upper respiratory tract specimens**

| Pathogens | Filmarray PP +  SOC/qPCR + | Filmarray PP +  SOC/qPCR - | Filmarray PP -  SOC/qPCR + | Filmarray PP -  SOC/qPCR - | PPA %  (95% CI) | NPA %  (95% CI) | OPA %  (95% CI) |
| --- | --- | --- | --- | --- | --- | --- | --- |
| Human rhinovirus | 24 | 1 | 1 | 34 | 96 (81-99) | 97 (86-100) | 97 (89-99) |
| Respiratory syncytial virus | 9 | 0 | 0 | 51 | 100 (70-100) | 100 (93-100) | 100 (94-100) |
| Adenovirus | 5 | 1 | 0 | 54 | 100 (57-100) | 98 (90-100) | 98 (91-100) |
| Parainfluenza virus | 2 | 1 | 0 | 57 | 100 (34-100) | 98 (91-100) | 98 (91-100) |
| Human metapneumovirus | 3 | 0 | 1 | 56 | 75 (30-95) | 100 (94-100) | 98 (91-100) |
| Influenza A virus | 2 | 0 | 0 | 58 | 100 (34-100) | 100 (94-100) | 100 (94-100) |
| Influenza B virus | 1 | 0 | 1 | 58 | 50 (9-91) | 100 (94-100) | 98 (91-100) |
| *Mycoplasma pneumoniae* | 4 | 0 | 0 | 56 | 100 (51-100) | 100 (94-100) | 100 (94-100) |
| All analytes | 50 | 3 | 3 | 424 | 94 (85-98) | 99 (98-100) | 99 (97-99) |

**Supplementary Table 6. Performance summary of the pathogens detected by the Filmarray pneumonia panel compared to standard-of-care testing and quantitative PCR of lower respiratory tract specimens**

| Pathogens | Filmarray PP +  SOC/qPCR + | Filmarray PP +  SOC/qPCR - | Filmarray PP -  SOC/qPCR + | Filmarray PP -  SOC/qPCR - | PPA %  (95% CI) | NPA %  (95% CI) | OPA %  (95% CI) |
| --- | --- | --- | --- | --- | --- | --- | --- |
| Human rhinovirus | 26 | 3 | 3 | 28 | 90 (74-96) | 90 (75-97) | 90 (80-95) |
| Respiratory syncytial virus | 8 | 0 | 1 | 51 | 89 (57-98) | 100 (93-100) | 98 (91-100) |
| Adenovirus | 3 | 2 | 0 | 55 | 100 (44-100) | 97 (88-99) | 97 (89-99) |
| Parainfluenza virus | 3 | 2 | 0 | 55 | 100 (44-100) | 97 (88-99) | 97 (89-99) |
| Human metapneumovirus | 4 | 0 | 0 | 56 | 100 (51-100) | 100 (94-100) | 100 (94-100) |
| Influenza A virus | 1 | 0 | 0 | 59 | 100 (21-100) | 100 (94-100) | 100 (94-100) |
| Influenza B virus | 1 | 0 | 0 | 59 | 100 (21-100) | 100 (94-100) | 100 (94-100) |
| *Mycoplasma pneumoniae* | 4 | 0 | 0 | 56 | 100 (51-100) | 100 (94-100) | 100 (94-100) |
| *Staphylococcus aureus* | 21 | 2 | 4 | 33 | 84 (65-94) | 94 (81-98) | 90 (80-95) |
| *Streptococcus pneumoniae* | 9 | 0 | 3 | 48 | 75 (47-91) | 100 (93-100) | 95 (86-98) |
| *Haemophilus influenzae* | 7 | 1 | 0 | 52 | 100 (65-100) | 98 (90-100) | 98 (91-100) |
| All analytes | 87 | 10 | 11 | 552 | 89 (81-94) | 98 (97-99) | 97 (95-98) |

**Supplement Figure 1. Potential pathogen distribution of upper and lower respiratory tract specimens from 60 children with severe community-acquired pneumonia.**

Supplement Figure 1 demonstrated the distribution of potential pathogen detected by Filmarray respiratory panel, pneumonia panel, standard-of-care diagnostic tests, and quantative polymerase chain reaction. The upper pie charts showed the pathogen distribution of viruses and *Mycoplasma pneumoniae* of upper and lower respiratory tract specimens. The lower pie charts revealed the pathogen distribution of bacteria of upper and lower respiratory tract specimens. Some microorganisms, such as cytomegalovirus, *Stenotrophomonas maltophilia*, and *Pneumocystis jirovecii*, were off-panel targets, shown by grayscale pie charts.
