# Supplementary material for: Application of nested multiplex polymerase chain reaction respiratory and pneumonia panels in children with severe community‐acquired pneumonia
Source: J Med Virol. 2022 Dec 2;95(1):e28334. doi: 10.1002/jmv.28334 (PMC10108056; doi:10.1002/jmv.28334)
Supplement: Supplementary file 2 — Supplementary information. [file JMV-95-0-s002.pdf]

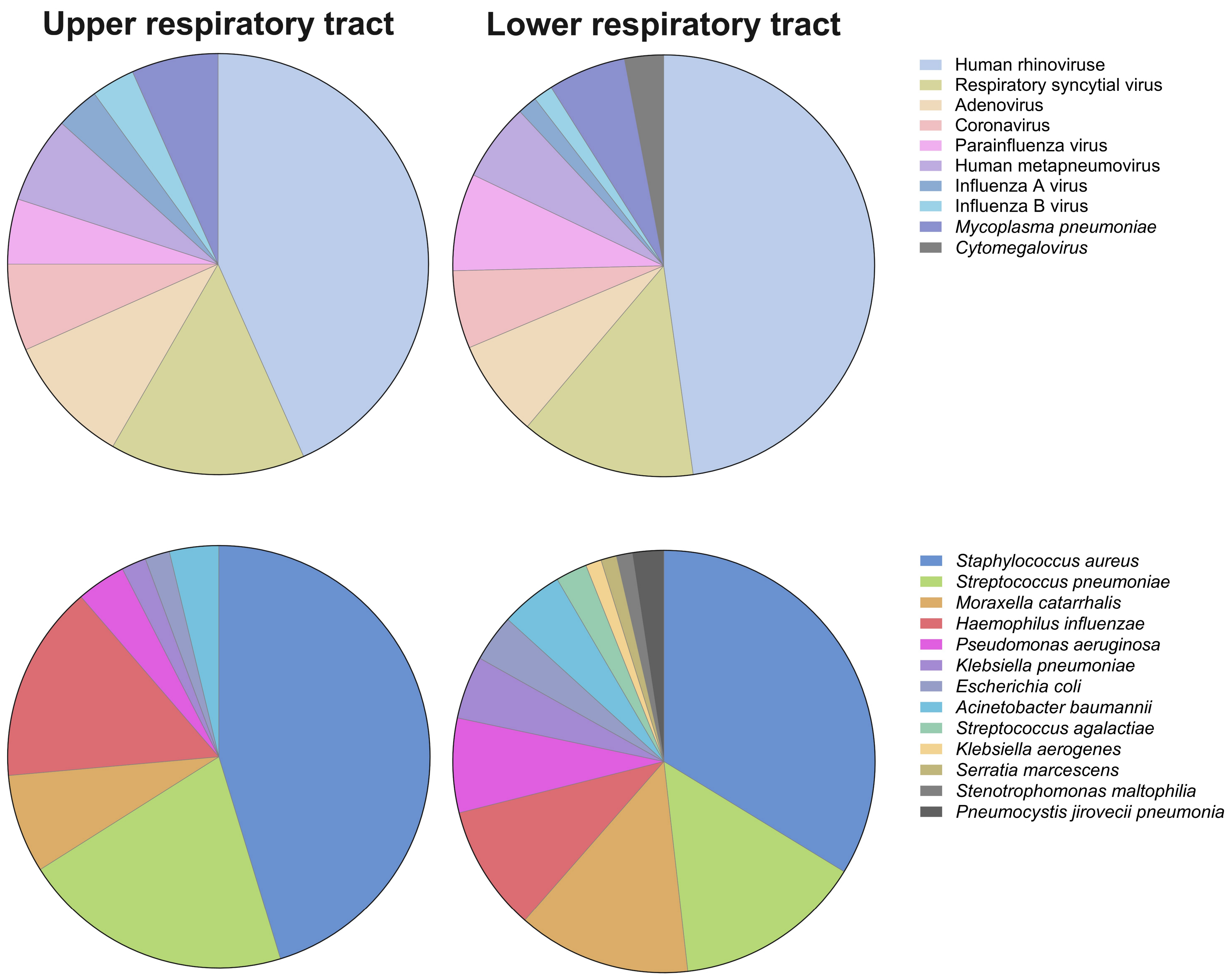
Upper respiratory tract
Lower respiratory tract
Human rhinoviruse
Respiratory syncytial virus
Adenovirus
Coronavirus
Parainfluenza virus
Human metapneumovirus
Influenza A virus
Influenza B virus
Mycoplasma pneumoniae
Cytomegalovirus
Staphylococcus aureus
Streptococcus pneumoniae
Moraxella catarrhalis
Haemophilus influenzae
Pseudomonas aeruginosa
Klebsiella pneumoniae
Escherichia coli
Acinetobacter baumannii
Streptococcus agalactiae
Klebsiella aerogenes
Serratia marcescens
Stenotrophomonas maltophilia
Pneumocystis jirovecii pneumonia
